# Multi-omics integrative modelling for stereotactic body radiotherapy in early-stage non-small cell lung cancer: clinical trial protocol of the MONDRIAN study

**DOI:** 10.1186/s12885-023-11701-9

**Published:** 2023-12-15

**Authors:** Stefania Volpe, Mattia Zaffaroni, Gaia Piperno, Maria Giulia Vincini, Maria Alessia Zerella, Federico Mastroleo, Federica Cattani, Cristiana Iuliana Fodor, Federica Bellerba, Tiziana Bonaldi, Giuseppina Bonizzi, Francesco Ceci, Marta Cremonesi, Nicola Fusco, Sara Gandini, Cristina Garibaldi, Davide La Torre, Roberta Noberini, Giuseppe Petralia, Lorenzo Spaggiari, Konstantinos Venetis, Roberto Orecchia, Monica Casiraghi, Barbara Alicja Jereczek-Fossa

**Affiliations:** 1 Division of Radiation Oncology, IEO European Institute of Oncology IRCCS, Milan, 20141 Italy; 2 Department of Oncology and Hemato-Oncology, University of Milan, Milan, 20122 Italy; 3 Department of Translational Medicine, University of Piemonte Orientale (UPO), Novara, 28100 Italy; 4 Unit of Medical Physics, European Institute of Oncology (IEO) IRCCS, Milan, 20141 Italy; 5 Department of Experimental Oncology, IEO European Institute of Oncology IRCCS, Milan, 20139 Italy; 6 Biobank for Translational and Digital Medicine, IEO, European Institute of Oncology IRCCS, Milan, Italy; 7 Division of Nuclear Medicine, IEO European Institute of Oncology IRCCS, Milan, 20141 Italy; 8 Unit of Radiation Research, IEO European Institute of Oncology, IRCCS, Milan, Italy; 9 Division of Pathology, IEO, European Institute of Oncology IRCCS, Milan, Italy; 10 SKEMA Business School, Université Côte d’Azur, Sophia Antipolis, France; 11 Precision Imaging and Research Unit, Department of Medical Imaging and Radiation Sciences, IEO European Institute of Oncology IRCCS, Milan, Italy; 12 Division of Thoracic Surgery, IEO, European Institute of Oncology IRCCS, Milan, 20141 Italy; 13 Scientific Directorate, IEO European Institute of Oncology IRCCS, Milan, 20141 Italy

**Keywords:** NSCLC, SBRT, ES-NSCLC, Radiomics, Genomics, Proteomics, NGS, Integrative model building, Clinical trial

## Abstract

**Background:**

Currently, main treatment strategies for early-stage non-small cell lung cancer (ES-NSCLC) disease are surgery or stereotactic body radiation therapy (SBRT), with successful local control rates for both approaches. However, regional and distant failure remain critical in SBRT, and it is paramount to identify predictive factors of response to identify high-risk patients who may benefit from more aggressive approaches. The main endpoint of the MONDRIAN trial is to identify multi-omic biomarkers of SBRT response integrating information from the individual fields of radiomics, genomics and proteomics.

**Methods:**

MONDRIAN is a prospective observational explorative cohort clinical study, with a data-driven, bottom-up approach. It is expected to enroll 100 ES-NSCLC SBRT candidates treated at an Italian tertiary cancer center with well-recognized expertise in SBRT and thoracic surgery. To identify predictors specific to SBRT, MONDRIAN will include data from 200 patients treated with surgery, in a 1:2 ratio, with comparable clinical characteristics. The project will have an overall expected duration of 60 months, and will be structured into five main tasks: (i) Clinical Study; (ii) Imaging/ Radiomic Study, (iii) Gene Expression Study, (iv) Proteomic Study, (v) Integrative Model Building.

**Discussion:**

Thanks to its multi-disciplinary nature, MONDRIAN is expected to provide the opportunity to characterize ES-NSCLC from a multi-omic perspective, with a Radiation Oncology-oriented focus. Other than contributing to a mechanistic understanding of the disease, the study will assist the identification of high-risk patients in a largely unexplored clinical setting. Ultimately, this would orient further clinical research efforts on the combination of SBRT and systemic treatments, such as immunotherapy, with the perspective of improving oncological outcomes in this subset of patients.

**Trial registration:**

The study was prospectively registered at clinicaltrials.gov (NCT05974475).

## Introduction

Lung cancer is the leading cause of cancer-related mortality, accounting for more than 257,000 deaths in Europe in 2020 [1]. Non-small cell lung cancer (NSCLC) is the most common type of lung cancer, encompassing diverse histological subtypes. Recent advances in screening initiatives, especially in high-risk populations- have yielded mortality benefits up to 20%: as a consequence, and for the first time, there has been an observed increase in the diagnosis of early-stage disease (ES-NSCLC). This has underscored the imperative to refine the understanding of tumor biology, in order to identify predictive and prognostic to orient clinical management [2].

Currently, those diagnosed with ES-NSCLC are potential candidates for either surgery or radiation therapy (RT) [3]. In the absence of robust evidence from randomized trials comparing the two treatment modalities [4–6], the choice of the optimal treatment is guided by clinical characteristics (such as pulmonary function), equipment availability, and the expertise of individual institutions. In Radiation Oncology, the advent of stereotactic body RT (SBRT) has significantly reshaped treatment paradigms for ES-NSCLC. A relevant aspect of such shift is the utilization of extremely hypofractionated schemes, and the delivery of higher biologically effective doses to the target, whose movements can be tracked in real-time across the respiratory phases. Other than being safe and well-tolerated, SBRT has a specific biological rationale, which consists in counteracting cancer cells repopulation through the induction of vascular damage, immune response, and alterations in the tumor microenvironment [7]. While achieving excellent rates of local control, regional and distant failures remain an issue [8]. Hence, the identification of individuals at higher risk for disease progression holds the potential to reduce progression and disease-related mortality, prevent overtreatment of low-risk patients, reduce avoidable healthcare expenditures and- ultimately- facilitate the transition to fully personalized treatment strategies in this clinical setting. This is especially relevant in the evolving landscape of combined approaches, including- as an example- the SBRT/immunotherapy combination.

Over the past decade, a more profound understanding of NSCLC immunobiology has led to the successful utilization of immune checkpoint inhibitors for locally-advanced and metastatic disease. While safety and efficacy in real-life scenarios are being tested [9, 10], the use of immunotherapy in early-stages has emerged as a hot topic in Thoracic Oncology [11]. Of note, trials are mainly investigating immunotherapy in surgical candidates (e.g. CHECKMATE 816 and KEYNOTE 671 trials), while a specific focus on SBRT is currently lacking [12, 13]. However, considering the well-recognized immunomodulatory effects of SBRT [14], its synergistic integration with immune checkpoint inhibitors holds the promise of enhancing the improvement of the oncological outcomes in poor SBRT responders. Thus, further investigations are mandatory.

To date, knowledge on potential predictors of response to SBRT is limited to a heterogenous set of clinical, radiological, metabolic and dosimetric parameters, stemming mainly from small, retrospective series [15, 16]. Other than these qualitative and semi-quantitative descriptors, the integration of high-throughput information (i.e., “omics”) holds the promise of providing novel predictive features that cannot be inferred from a priori clinical knowledge (e.g. a radiomic signature of radioresistance). Among these -omics approaches, radiomics stands out as the most easily scalable, thanks to both the wide imaging availability and low implementation costs. In contrast, translational approaches- such as genomics- are by far less common. Additionally, the integration of data from different information layers (e.g. radiomics and genomics) could further enhance outcome prediction compared to the use of individual information layers alone [17]. In essence, the rationale behind the integration of multi-dimensional information is that each histopathological, radiological, genomic, transcriptomic feature- whether qualitative, semiquantitative, or purely quantitative, represents the piece of a larger and more intricated jigsaw. Nonetheless, whether the combination of multiple information layers really translates into improved outcome prediction, remains an open question.

## Methods/ design

### Aim and setting of the study

The primary aim of this study- named MONDRIAN (Multi-Omics Integrative Modeling for Stereotactic Body Radiotherapy in Early-stage Non-Small Cell Lung Cancer), is to identify a multi-omic biomarker of response to SBRT in ES-NSCLC. Additionally, the study aims to assess whether the combination of clinical, imaging and biologically-derived information could yield better prediction than individual-omics alone. This will be realized through a data-driven, bottom-up approach integrating prospectively collected data from the radiomic, genomic, and protein domains, to more consolidated clinical parameters, as detailed below.

MONDRIAN is designed as a monocentric, prospective observational explorative cohort trial. It is expected to enroll ES-NSCLC patients treated with either SBRT or surgery with a 1:2 ratio, respectively, for a total of 300 patients, over a 5-year timeframe. The primary endpoint is progression-free survival (PFS) defined as the time between the beginning of SBRT or the date of surgery, and any disease progression (local, regional, distant), death from any cause, or last follow-up, whichever comes first. Secondary endpoints are the investigation of prognostic factors from clinical, imaging, genomic and proteomic data, the longitudinal validation of gene expression and proteomics profiling among tissue- and liquid biopsies-derived samples, and the creation of a permanent dataset of high-quality, prospectively collected multi-dimensional data.

The whole study will be conducted at the European Institute of Oncology (Istituto Europeo di Oncologia, IEO) IRCCS, in Milan, Italy.

### Study population

Inclusion criteria are as follows:

- Age ≥ 18 years;
- Histological diagnosis of NSCLC; all histologies will be considered eligible (e.g. squamous, adenocarcinoma, large cell carcinoma);
- American Joint Committee on Cancer Tumor Node Metastasis (AJCC TNM) classification (8th Edition) clinical stage I-II;
- Eastern Cooperative Oncology Group (ECOG) performance status 0–1;
- Ability and willingness to sign a written informed consent for treatment and study participation.
- No diagnosis of other invasive cancer (within 3 years before the diagnosis of ES-NSCLC);
- No absolute contraindications to either surgery (e.g. severe comorbidities) or SBRT (e.g. active connective diseases, severe pulmonary fibrosis);
- No mental diseases or psychiatric disorders that cannot ensure the acquisition of a valid informed consent.

### Study design

The study is divided into five main tasks, summarized in Table 1. Of note, being an observational trial, the care path will strictly adhere to best clinical practice guidelines [3], and no additional examinations will be required with the sole exception of a venous blood sampling, which will be performed at baseline and at the first disease progression or last follow-up (whichever comes first), as an optional part of the study. The aim of this sub-task is to perform a longitudinal validation of gene expression and proteomic profiling between tissue samples and liquid biopsies. Due to the experimental nature of these analyses, along with their potentially high risk/high gain profile- this sub-study is considered as a benchmark, and participation will be limited to 60 patients treated with SBRT. Participation in this sub-study will be optional, and declining will not affect enrollment in the main study.

**Table 1:** Summary of the five tasks of the study

Task	Timeframe (months)	Task Description
Task 1- Clinical Study	1–48	Eligibility check and patient enrollment (WP1), collection of baseline patient-, disease- and treatment-related data (WP2), follow-up and collection of patient-, disease and treatment-related data (WP3)
Task 2- Imaging/Radiomic Study	1–60	Collection of qualitative and semi-quantitative CT image metrics (WP4), collection of qualitative and semi-quantitative 18FDG-PET/CT image metrics (WP5), radiomic analysis on CT and [18 F]-FDG-PET/CT images (WP6)
Task 3- Gene Expression Study	1–48	Sample collection and storage of FFPE samples from either the resected tumor or tissue biopsy (WP7), gene expression profiling through the NGS-based assay OIRRA (WP8)
Task 4- Proteomic Study	1–48	Sample collection and storage of FFPE samples from either the resected tumor or tissue biopsy (WP9), proteomics analysis through the PATH-MS method (WP10)
Task 5- Integrative Model Building	48–60	Data cleaning, exploratory data analysis, regularization (WP11), model training and validation (WP12)

**Abbreviations**: [18 F]-FDG-PET/CT: [18 F]Fluorodeoxyglucose positron emission tomography, CT: computed tomography, FFPE: formalin-fixed paraffin-embedded, OIRRA: Oncomine™ Immune Response Research Assay, PATH-MS: (PAThology tissue analysis of Histones by Mass Spectrometry, WP: work package

**Fig. 1:**
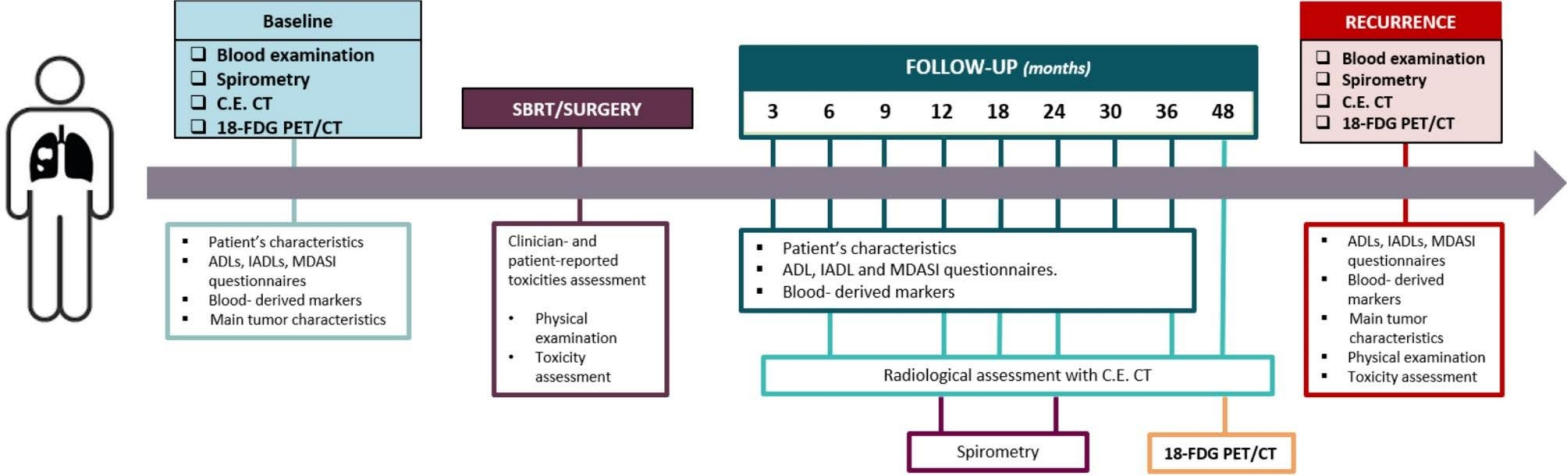
Schematic representation of Task 1. **Abbreviations**: [18 F]-FDG-PET/CT: [18 F]Fluorodeoxyglucose positron emission tomography, ADL: Activities of Daily Living, C.E. CT: contrast-enhanced computed tomography, IADL: Instrumental Activities of Daily Living, MDASI: MD Anderson Symptom Inventory, SBRT: Stereotactic Body Radiotherapy

A detailed overview of the clinical Task 1 is provided in Fig. 1. Specifically, patient-, disease- and treatment-related data will be collected for both cohorts at baseline and during the course of follow-up, as indicated below:

- Patient’s characteristics: demographics, performance status, comorbidities (individual and per the Charlson Comorbidity Index), smoking status, pack-years, and body mass index.
- Patient-reported outcomes questionnaires (e.g. Activities of Daily Living- ADLs- and Instrumental Activities of Daily Living- IADLs- questionnaires).
- Pulmonary function, assessed by spirometry.
- Blood-derived inflammation and nutritional markers, including complete blood count and derived parameters (e.g. neutrophil/ lymphocytes ratio), renal and hepatic function, albumin, lactic dehydrogenase.

Main tumor characteristics including overall staging and lesion location will be collected, as well. This qualitative information will be complemented by the inclusion of qualitative and semiquantitative parameters Task 2. Finally, treatment characteristics will be retrieved for both treatment cohorts. In the case of SBRT, beyond the prescribed total dose, clinically meaningful dose-volume statistics will be considered (i.e. maximum dose, minimum dose, mean dose, and percentage volume receiving greater than or equal to the prescription dose). Alongside punctual dose parameters, the area under the dose-volume histogram curve will also be evaluated to better assess dose distribution to both the target volume and to the nearby healthy structures.

### Treatment modalities

Following multidisciplinary discussion within the institutional lung tumor board, either SBRT or surgery will be indicated [18]. Considering the former, treatment will be delivered in three or five fractions, considering an α/β ratio of 10 Gy, as supported by available evidence [3, 19, 20]. The choice of the optimal fractionation schedule will be made according to clinical parameters, including tumor size, location (central vs. peripheral) and preexisting patient’s comorbidities (e.g. chronic obstructive pulmonary disease, interstitial disease lung). Treatment planning will be realized with dedicated systems, namely Raystation [RaySearch Laboratories] and Precision-Cyberknife [Accuray], while treatment delivery will be performed with either TrueBeam [Varian], Tomotherapy [Elekta], or Cyberknife [Accuray]. Regarding surgery, all patients will receive anatomical pulmonary resection such as segmentectomy or lobectomy with systematic lymphadenectomy, in compliance with current clinical practice guidelines [3, 21].

### Power calculations

The main aim of the MONDRIAN study is to assess the predictive value of the multi-omic predictive score for SBRT response, with the primary outcome of interest being PFS. A sample size of 270 allows to verify whether the score (classified as low and high, based on the median value) is able to predict treatment response on PFS with 80% of power [22]. With a median progression-free survival time (MPFST) of 27 and 14 months in the SBRT and surgery groups, respectively [23], for patients with high score, the corresponding hazard ratio (HR) is 0.52. For patients with the low score, we assume a MPFSTs of 14 and 27 months in the SBRT and surgery groups, respectively, with a corresponding HR of 1.93. Under the assumption of uniform distribution for the censoring time during the study period, a two-sided 1% type I error calculation was set to account for multiple testing. Given the possibility of 10% of missing values/lost to follow-up, an approximate number of 300 patients, with a 1:2 ratio for SBRT and surgery, respectively, will be enrolled.

### Ethical considerations, funding and registration

The study will comply with the Declaration of Helsinki and with the principles enunciated by the International Conference on Harmonization and Good Clinical Practice in May 1996 [24, 25]. Signature of the general written informed consent for study participation will be required to all participants. An additional written informed consent will be provided to SBRT candidates in case they are willing to be enrolled in the sub-study for the longitudinal validation of non-invasive genomic and proteomic signatures, as detailed above.

The MONDRIAN trial is supported by a research grant from the Italian Association for Cancer Research (Associazione Italiana per la Ricerca sul Cancro, AIRC)- Next Generation Clinician Scientist- Call 2022 (ID: 28,282).

The study has been approved by the Institutional Ethical Committee (ID number: IEO 1973- UID3940), and then registered at clinicaltrials.gov (NCT05974475).

## Discussion

The rationale of the MONDRIAN study fits into the early stages of applying the omics sciences to Radiation Oncology, and into the wider concepts of personalized medicine. These principles are applied to the largely uninvestigated setting of ES-NSCLC, with the primary aim of investigating factors associated with radiosensitive and radioresistant phenotypes. In addition to seeking a mechanistic understanding of the disease, the study aspires to uncover clinically actionable insights by the identification of different risk profiles for disease progression. Arguably, this would set a benchmark for further investigations on treatment intensification strategies for high-risk patients, which may include dose escalation and/or combination therapies (e.g. immunotherapy).

Several risk mitigation strategies have been developed to ensure sufficient enrollment, accurate prospective collection of reliable data, and- overall- successful study completion. Of these, it is important to underline that interdependence among tasks will be significantly diminished thanks to the training of single- and dual-omics models, in addition to the multi-omic model. This strategy serves two purposes: on the one hand, it minimizes the weight of potentially uninformative data, on the other, it allows to explore how the combination of omic-features contributes to the predictive performance of models.

Another potentially relevant issue may involve negative patient selection in the SBRT cohort. Indeed, SBRT is often regarded as the second-choice curative treatment for ES-NSCLC, particularly in case of contraindications to anesthesia and/or surgery. Consequently, a certain degree of selection bias is anticipated, also due to the non-randomized nature of the MONDRIAN study. Hence, all models will be adjusted using a propensity score, which will be calculated for each patient by combining the beta coefficients of the clinical variables included in a logistic regression model where the treatment (SBRT vs. surgery) serves as the outcome. This adjustment aims to minimize the impact of potential confounders and ensure a reasonable level of comparability between treatment groups. A propensity score approach based on patient matching will also be employed and compared with the method relying on propensity score adjustment.

To the best of our knowledge, this is the first study to investigate predictors of response to SBRT in ES-NSCLC through the integration of data from multiple -omic disciplines. This would provide an unprecedented opportunity to characterize the disease, and to elucidate the interaction between determinants of radiosensitivity and radioresistance. The focus on potentially actionable targets is timely in the wider context of high-precision RT, RT/systemic therapy combinations, and personalized medicine.

## Data Availability

Not applicable.

## List of abbreviations

ADLs: Activities Of Daily Living
AIRC: Associazione Italiana Per La Ricerca Sul Cancro
AJCC TNM: American Joint Committee On Cancer Tumor Node Metastasis Classification
ECOG: Eastern Cooperative Oncology Group
ES-NSCLC: Early-Stage Non-Small Cell Lung Cancer
FFPE: Formalin- Fixed Paraffin Embedded
HR: Hazard Ratio
IADLs: Instrumental Activities Of Daily Living
MDASI: MD Anderson Symptom Inventory
MPFST: Median Progression-Free Survival Time
NSCLC: Non-Small Cell Lung Cancer
NGS: Next Generation Sequencing
OIRRA: Oncomine Immune Response Research Assay
PATH-MS: Pathology Tissue Analysis Of Histones By Mass Spectrometry
PET/CT: Positron Emission Computed Tomography
PFS: Progression-Free Survival
RT: Radiation Therapy
SBRT: Stereotactic Body Radiation Therapy
WP: Work Package
